# High Levels of Galectin-3 and Uric Acid Are Independent Predictors of Renal Impairment in Patients with Stable Coronary Artery Disease

**DOI:** 10.3390/jcm14155264

**Published:** 2025-07-25

**Authors:** Nayleth Leal-Pérez, Luis M. Blanco-Colio, José Luis Martín-Ventura, Carlos Gutiérrez-Landaluce, Ignacio Mahíllo-Fernández, María Luisa González-Casaus, Óscar Lorenzo, Jesús Egido, José Tuñón

**Affiliations:** 1 Department of Gynecology and Obstetrics, Príncipe de Asturias University Hospital, 28805 Madrid, Spain; nayleth@gmail.com; 2 Laboratory of Vascular Pathology, IIS-Fundación Jiménez Díaz, 28040 Madrid, Spain; lblanco@fjd.es (L.M.B.-C.); jlmartin@fjd.es (J.L.M.-V.); jegido@quironsalud.es (J.E.); 3 Biomedical Research Networking Center on Cardiovascular Diseases (CIBERCV), 28029 Madrid, Spain; 4 Department of Medicine, Medical School, Autónoma University, 28049 Madrid, Spain; olorenzo@fjd.es; 5 Department of Cardiology, Hospital Universitario de Fuenlabrada, 28942 Madrid, Spain; cgutierrezl@salud.madrid.org; 6 Research Unit, IIS-Fundación Jiménez Díaz, 28040 Madrid, Spain; imahillo@fjd.es; 7 Laboratory of Nephrology and Mineral Metabolism, Hospital La Paz, 28046 Madrid, Spain; mlgcasaus@gmail.com; 8 Renal, Vascular and Diabetes Research Laboratory, IIS-Fundación Jiménez Díaz, 28040 Madrid, Spain; 9 Diabetes and Associated Metabolic Diseases Networking (CIBERDEM), 28029 Madrid, Spain; 10 Department of Cardiology, IIS-Fundación Jiménez Díaz, 28040 Madrid, Spain

**Keywords:** coronary artery disease, Galectin-3, glomerular filtration rate, uric acid

## Abstract

**Background:** High plasma levels of Galectin-3 (Gal-3) and uric acid (UA) are associated with a decline in renal function in different populations. However, this association has not yet been studied in patients with coronary artery disease (CAD). **Methods:** We included 556 patients with stable CAD. Plasma levels of Gal-3, UA, N-Terminal probrain natriuretic peptide (NT-proBNP), calcidiol, fibroblast growth factor 23, phosphate, parathormone, and klotho were assessed at baseline. The primary outcome was the percentage decrease in eGFR; the secondary outcomes were the absolute decrease in eGFR and achieving a reduction of ≥20% in this parameter. **Results:** Age was 63.1 ± 12.2 years, and 73.9% of patients were male. The median eGFR was 86.77 (72.27, 97.85) mL/min/1.73 m^2^. After 3.47 (2.10–5.72) years of follow-up, eGFR declined by 3.62% [−2.07–13.82]. Baseline UA (0.012 [CI95% 0.003, 0.020]; *p* = 0.008), Gal-3 (0.0153 [CI95% 0.001, 0.029]; *p* = 0.037), and NT-proBNP (0.017 [CI95% 0.000–0.025]; *p* = 0.027) were independent positive predictors of the percentage decrease in eGFR, while calcidiol (−0.005 [CI95% −0.009, −0.002]; *p* = 0.005) was an inverse predictor of this outcome. Similarly, UA and Gal-3 were positive independent predictors of the absolute decline in eGFR (0.009 [0.003, 0.017]; *p* = 0.004 and 0.012 [0.001, 0.023]; *p* = 0.031, respectively), while calcidiol was inversely associated (−0.003 [−0.005]–[−0.001]; *p* = 0.020). Uric acid (1.237 [1.046–1.463]; *p* = 0.013) and NT-proBNP (1.000 [1.000–1.001]; *p* = 0.049) levels were positive independent predictors of a ≥20% decrease in eGFR. In patients with eGFR ≥ 60 mL/min/1.73 m^2^, UA was the only biomarker independently associated with renal function decline. **Conclusions:** In patients with CAD and normal or mildly reduced renal function, UA and Gal-3 plasma levels are independent positive predictors of a future decrease in eGFR. These findings could lead to a change in the approach to patients with CAD in the future.

## 1. Introduction

Chronic kidney disease (CKD) is a major health concern, affecting 13.4% (11.7–15.1%) of the global population [1]. It is considered a risk factor for cardiovascular disease [2] and is directly associated with increased morbidity and mortality worldwide [3,4]. There is an association between CKD and coronary artery disease (CAD), given that atherosclerosis may affect both organs [5]. Identifying independent predictors of renal damage in CAD patients could improve patient management, enabling timely intervention strategies to mitigate disease progression.

Galectin-3 (Gal-3) has been suggested to play a role in renal damage, primarily by promoting fibrosis [6,7]. Consequently, Gal-3 plasma levels have been shown to predict a decrease in estimated glomerular filtration rate (eGFR) in various settings, including patients with CKD [8], diabetes [9], and the general population [10,11], among others. However, it remains unclear whether Gal-3 levels can predict an impairment of renal function in CAD patients.

Increased uric acid (UA) plasma levels have been associated with the development and progression of CKD and cardiovascular disease [12,13,14]. While plasma UA peaks have historically been considered a consequence of renal failure, new theories challenge this perspective, suggesting that UA may also be an active contributor to kidney damage [13]. Uric acid has been suggested to mediate renal damage by promoting inflammation [15] and activating the renin–angiotensin–aldosterone system [16,17], among other biological processes. The potential of UA as a marker of future renal function deterioration has been studied in different population subgroups, yielding controversial results in CKD patients [18,19,20,21,22,23,24] and positive results in the general population [25,26,27,28,29,30], patients with hypertension [31], and patients with type I [32] and type 2 diabetes mellitus [33]. However, no results have been reported on the potential predictive role of UA plasma levels in patients with CAD.

In this study, we investigate the potential role of Gal-3 and UA plasma levels in predicting a decrease in eGFR in patients with stable CAD. We also examine several components of mineral metabolism (fibroblast growth factor 23 [FGF-23], α-Klotho, 25-dihydroxyvitamin D [calcidiol], phosphate, and parathyroid hormone [PTH]), as abnormalities in these factors have been associated with a decline in renal function [34]. Furthermore, we determined N-terminal pro-brain natriuretic peptide (NT-proBNP), which is associated with heart failure and cancer [35].

## 2. Materials and Methods

### 2.1. Patients

The present study is a sub-study of the BACS and BAMI studies (biomarkers in acute coronary syndrome and biomarkers in acute myocardial infarction), which were conducted in five hospitals in Madrid, as previously described [36].

Six months after experiencing the index acute coronary syndrome, patients attended an outpatient consultation. At this time, clinical variables were recorded, and blood samples were taken.

Of the 1257 patients included during the acute event, 964 attended the outpatient appointment and completed the follow-up. The present study focuses on the 556 patients included in two of the five centeres (Fundación Jiménez Díaz and Puerta de Hierro), as these were the only institutions with analytical data available at the end of the follow-up period.

### 2.2. Ethics Statement

The research protocol follows the ethical guidelines outlined in the Declaration of Helsinki of 1975, having received prior approval from the ethics committees of the institutions participating in this research: the Fundación Jiménez Díaz and Puerta de Hierro Hospitals. All patients provided voluntarily informed consent and signed all relevant documents.

### 2.3. Study Design

Plasma extraction and baseline visits took place between January 2007 and December 2014. The last follow-up visits were carried out in June 2016. Creatinine values at the end of the follow-up were obtained from the electronic records of the patients. We used the chronic kidney disease epidemiology collaboration (CKD-EPI) equation to estimate eGFR. The primary outcome was the percentage decrease in eGFR from baseline to the end of follow-up. As secondary endpoints, we assessed (a) the decrease in eGFR in absolute numbers and (b) a reduction of 20% or more in eGFR from baseline to the end of follow-up. Traditional markers of renal impairment, such as doubling of creatinine levels or development of end-stage kidney disease, which are commonly used in populations with CKD, were not included as endpoints. This is because it was not expected that a significant number of patients with normal or mildly reduced renal function would develop these outcomes. Finally, we also analyzed the incidence of acute ischemic events (acute coronary syndrome, ischemic stroke, or TIA), heart failure, and death.

### 2.4. Biomarker and Analytical Studies

Plasma determinations were performed at the Vascular Pathology and Biochemistry laboratories at Fundación Jiménez Díaz and at Nephrology department of the Gómez-Ulla hospital for mineral metabolism. UA determinations were performed at the Biochemistry laboratories of the Fundación Jiménez Díaz and Puerta de Hierro hospitals. The investigators who performed the laboratory studies were unaware of the clinical data. Twelve-hour fasting venous blood samples were withdrawn and collected. Blood samples were centrifuged at 2500 g for 10 min, and plasma was stored at −80 °C. UA, lipids, glucose, and creatinine determinations were performed by standard methods (ADVIA 2400 Chemistry System, Siemens Healthineers, Munich, Germany). Plasma calcidiol levels were quantified by chemiluminescent immunoassay (CLIA) on the LIAISON XL analyzer (LIAISON 25OH-Vitamin D total Assay DiaSorin, Saluggia, Italy), FGF23 was measured by an enzyme-linked immunosorbent assay, which recognizes epitopes within the carboxyl-terminal portion of FGF-23 (Human FGF23, C-Term, Immutopics Inc., San Clemente, CA, USA), klotho levels were determined by ELISA (Human soluble alpha klotho assay kit, Immuno-Biological Laboratories Co., Gunma, Japan), the intact parathyroid hormone was analyzed by a second-generation automated chemiluminescent method (Elecsys 2010 platform, Roche Diagnostics, Mannheim, Germany), phosphate was determined by an enzymatic method (Integra 400 analyzer, Roche Diagnostics, Mannheim, Germany), high-sensitivity C-reactive (hs-CRP) protein was assessed by latex-enhanced immunoturbidimetry (ADVIA 2400 Chemistry System, Siemens Healthineers, Munich, Germany), and NTproBNP was determined by immunoassay (VITROS; Ortho Clinical Diagnostics Raritan, NJ, USA). Galectin-3 was determined using commercially available enzyme-linked immunoabsorbent assay kits (DCP00, R&D Systems, Minneapolis, MN, USA), and Hs-cTnI was assessed by direct chemiluminescence (ADVIA Centaur; Siemens Healthineers, Berlin, Germany). All experiments were performed in duplicate.

### 2.5. Statistical Analysis

Quantitative data following a normal distribution are presented as the mean ± standard deviation (SD); those not normally distributed are displayed as a median (interquartile range). Qualitative variables are presented as percentages.

The influence of baseline variables, as displayed in Table 1, on outcome variables was first assessed using simple linear regression analysis for numerical dependent variables and simple logistic regression for qualitative dependent variables. Subsequently, multiple linear regression and multiple logistic regression analyses were performed, entering the variables that achieved a “*p*”-value < 0.20 during univariate analyses. Furthermore, age, sex, Caucasian race, body mass index, and hypertension were included in multivariate analyses, independently of the “*p*”-value obtained during univariate analyses. Cox univariate and multivariate analyses were used to investigate the potential predictors of adverse cardiovascular events. A power analysis for the global F-test was conducted, using the effect size f^2^ derived from the obtained R^2^ [37].

Analyses were performed with R v3.5.1 (R Foundation for Statistical Computing, Vienna, Austria). Statistical significance was set at *p* < 0.05 (two-tailed).

## 3. Results

### 3.1. Patients

The median follow-up period was 3.47 (2.10–5.72) years. The median age was 63.1 ± 12.2 years; the majority of patients were male (73.9%) and of Caucasian ethnicity (95.1%) (Table 1). Sixty-five percent had hypertension, and 22.7% had diabetes. In total, 4.1% were receiving hypouricemic agents (Alopurinol). The median eGFR was 86.77 (72.27, 97.85) mL/min/1.73 m^2^, and 7.6% of the patients had a left ventricular ejection fraction lower than 40%.

The index event, prior to the start of this study, was ST-elevation myocardial infarction in 326 and non-ST elevation acute coronary syndrome in 230. Seventy-five percent of all patients underwent complete revascularisation. The interval between the index event and the outpatient blood extraction for this substudy was 6.2 months (6.1–6.6).

### 3.2. eGFR Percentage Decline During Follow-Up

Only nine (1.6%) patients doubled their plasma creatinine levels, and one patient started haemodialysis at the end of the follow-up. The estimated glomerular filtration rate declined by 3.62% [−2.07–13.82]. To detect predictors of the percentage decrease in eGFR, a simple linear regression analysis was first performed (see Supplementary Table S1 online). Then, a multiple linear regression analysis was conducted, which showed that elevated plasma levels of UA, Galectin-3, and NT-proBNP, as well as age, the presence of hypertension, and the use of insulin, among others, were independently and directly related to the percentage decrease in eGFR (Table 2). On the other hand, baseline calcidiol, male gender, and the use of anticoagulants were inversely associated with this outcome.

A power analysis for the global F-test, using the effect size f^2^ derived from an R^2^ ≈ 0.13 showed that with the 13 predictors obtained, an *n* ≈ 500 and an α error = 0.05, the resulting power exceeds 99%.

When UA and Galectin-3 were removed from the model, R^2^ decreased to 0.105 (Supplementary Table S2 online).

### 3.3. Absolute Difference in the Whole Population Between eGFR at Baseline and at the End of Follow-Up

The glomerular filtration rate diminished from 86.77 (72.27, 97.85) to 82.15 (64.39, 95.00) mL/min/1.73 m^2^ (*p* < 0.001) during follow-up, resulting in a median decrease of 4.62 mL/min/1.73 m^2^ during 3.47 years of follow-up (1.33 mL/min/1.73 m^2^ per year). Table S3 in the Supplementary Material shows the variables predicting this outcome at univariate analysis.

During the multiple linear regression analysis, UA and Galectin-3 plasma levels, age, the existence of hypertension, and treatment with insulin were again directly related to the decrease in eGFR during follow-up (Table 3).

On the other hand, calcidiol, and the use of anticoagulants were inversely related to this outcome.

### 3.4. Individual Falls of 20% or More in the Glomerular Filtration Rate During Follow-Up

The eGFR decreased by 20% or more in 82 patients. This represents 14.7% of the whole sample and 22.8% of the 360 patients who experienced a fall in eGFR. To detect probable predictors of this reduction, we performed a simple logistic regression analysis. Table S4 of the Supplementary Material shows the predictors of this outcome. Multiple logistic regression analysis showed that UA levels, as well as NT-proBNP, the existence of hypertensionand the use of nitrates and anticoagulant therapy were directly and independently associated with this outcome (Table 4).

### 3.5. Absolute Differences Between eGFR at Baseline and the End of Follow-Up According to the Presence of Significant CKD

We estimated the determinants associated with the absolute decrease in eGFR at follow-up in patients with and without eGFR < 60 mL/min/1.73 m^2^ at baseline. In total, 72 patients (12.9%) had an eGFR < 60 mL/min/1.73 m^2^, and 484 patients (87.1%) had an eGFR ≥ 60 mL/min/1.73 m^2^. The results of the simple logistic regression analysis are displayed in Tables S5 and S6 of the Supplementary Material. During the multiple linear regression analysis, in patients with eGFR ≥ 60 mL/min/1.73 m^2^, UA, but not Gal-3, was independently and positively associated with a decrease in eGFR, along with age, the existence of hypertension and the prescription of insulin, while caucasian race and male gender were inversely related to this outcome (Table 5). In patients with eGFR < 60 mL/min/1.73 m^2^, calcidiol was the only biomarker that was an independent predictor of the absolute decrease in eGFR, showing an inverse relationship.

### 3.6. Influence of Baseline Variables on the Appearance of Cardiovascular Events

During follow-up, 91 patients (16.4%) developed a major cardiovascular event (acute ischemic events (i.e., acute coronary syndrome, ischemic stroke, or TIA), heart failure, or death). From them, 15 patients had two events, 13 had three events, and 4 developed four events. In total, there were 20 episodes of unstable angina, 7 acute myocardial infarctions with ST elevation, 20 acute myocardial infarctions without ST elevation, 36 episodes of heart failure, 9 ischemic strokes, 12 transient ischemic attacks, and 40 deaths. The cause of death was cardiovascular in 18 cases (3.2%), oncological in 8 (1.4%), infection in 3 (0.5%), renal failure in 1 (0.2%), pancreatitis in 2 (0.4%), gastrointestinal bleeding in 1 (0.2%, other in 3 (0.5%), and unknown in 4 (0.7%).

Several variables were related to the prediction of events during the univariate analysis (Supplementary Table S7). However, during the multivariate analysis, only NT-ProBNP and PTH plasma levels were independent predictors of major cardiovascular events in our cohort, along with different clinical variables (Table 6). Neither UA levels nor Gal-3 or calcidiol were independently associated with the occurrence of this outcome.

## 4. Discussion

Galectin-3 levels have been associated with the progression of CKD in different cohorts, including the general population [10,11], patients with CKD [38,39], and diabetes [9], among others. However, no studies have evaluated this in patients with CAD. This is therefore the first report to show that Gal-3 is an independent predictor of a decline in renal function in this population. This is relevant because atherosclerosis is the main cause of CAD, and it may also contribute to CKD [40]. Furthermore, the extensive adjustment for many clinical and analytical variables, including the components of the mineral metabolism system, reinforces the value of Gal-3 as an independent predictor of renal function.

The main pathway through which Gal-3 may be involved in kidney damage is the promotion of fibrosis, as evidenced at an experimental level [6,7,41]. However, Gal-3 inhibition has not been demonstrated to decrease collagen markers in patients with hypertension [41], and further research is required to confirm whether this molecule may be a potential target to prevent renal damage in humans.

The role of UA plasma levels as a predictor of worsening renal function and cardiovascular events has been controversial. While several studies conducted primarily on CKD patients have failed to establish an association between UA levels and renal function decline [18,19,20,21,22,23], other research involving a healthy population [25,26,27,28,29,30], CKD stage 3–4 patients [23,24], individuals with hypertension [30], IgA nephropathy [42], and diabetes mellitus [32,33] has revealed a positive association. Finally, the results in patients receiving a kidney transplant are diverse [43].

To our knowledge, no previous studies have investigated the potential association between UA levels and worsening renal function in patients with CAD and normal or mildly reduced renal function. In the present study, we demonstrate that high plasma UA levels are independent predictors of a worsening in the eGFR in this population. This result was evident when eGFR was analyzed in different ways and corrected for an extensive set of variables, including demographic data, cardiovascular risk factors, previous cardiovascular history, drug treatment, and a wide range of analytical variables, such as NT-proBNP, mineral metabolism components, hs-CRP, and lipids. Interestingly, the adjustments performed in most of the above-described studies were less extensive than those performed in the present work. In our study, the median eGFR decreased from 86.77 to 82.15. This equates to a reduction in eGFR of 4.62 mL/min/1.73 m^2^ over 3.47 years of follow-up, which amounts to a yearly decrease of 1.33 mL/min/1.73 m^2^. Interestingly, the annual decline in renal function in the general population is approximately 1 mL/min/1.73 m^2^ [44], indicating that the decline in eGFR in our CAD patients is comparable to that observed in the general population. Of interest, most of our patients had single-vessel disease and a low prevalence of previous peripheral arterial disease. Therefore, we cannot exclude the possibility that eGFR decline may be faster in populations with more extensive atherosclerosis.

Finally, UA remained a powerful independent predictor of renal function decline in the subgroup of patients with eGFR ≥ 60 mL/min/1.73 m^2^. The absence of significant predictive power in patients with eGFR < 60 mL/min/1.73 m^2^ is probably related to the small size of this subgroup (72 patients).

Among the mechanisms through which UA could affect renal function, the overactivation of the renin–angiotensin–aldosterone system [16,17] and vascular inflammation [15] have been proposed.

In contrast to the observed worsening of renal function, UA and Gal-3 plasma levels were not independent predictors of the incidence of cardiovascular events per se. Previous studies have shown that UA plasma levels are independent predictors of cardiovascular events, particularly in patients with heart failure [43], myocardial infarction [45], and even in the general population [46,47]. However, adjustments were not made for mineral metabolism components [48]. Regarding Gal-3, we obtained conflicting results [36,49]. In this study, only PTH and NT-proBNP levels were independent predictors of the incidence of cardiovascular events. Beyond the well-known prognostic value of NT-proBNP as a prognostic biomarker [50], PTH also has predictive ability [51,52]. Furthermore, PTH has been linked to the pathophysiology of heart failure and left ventricular hypertrophy [53,54], although not all the studies confirm this relationship [55,56]. Currently, there is no consensus on the relationship between PTH and CAD. Thus, PTH levels have been linked to the incidence and progression of CAD [57,58] and the incidence of cardiovascular events and mortality [59,60]. Conversely, other studies have failed to confirm this association [61].

Some limitations of this study must be acknowledged. Firstly, urinary albumin excretion, which is related to renal function, was not assessed because urine samples were not collected. While a comprehensive assessment of renal function includes both eGFR and the urine albumin-to-creatinine ratio, the latter was not available at the time of the patient’s recruitment. Without these data, the results cannot be fully interpreted or compared with those of other studies. Furthermore, our work is based on a single biomarker assessment, and further determinations during the follow-up period could have provided additional relevant information. Secondly, the fact that 95.1% of patients were Caucasian limits the applicability of our findings to more ethnically diverse populations. Thirdly, the existence of renal artery stenosis was not studied, which could have influenced the evolution of renal function in our patients. Finally, the low R^2^ values in the models presented in Table 2 and Table 3 of the results (0.126 and 0.115, respectively) indicate that the models only explain 12.6% and 11.5%, respectively, of the obtained outcomes. Clarifying this point is essential for a better understanding of these results. Nevertheless, the strength of our work lies in adjusting for multiple clinical and analytical variables to correct for confounding bias. Due to these limitations, our results should be considered preliminary and require further validation in future studies.

## 5. Conclusions

In conclusion, this study shows that high UA and Galectin-3 levels are independent predictors of eGFR decline in patients with CAD and normal or mildly reduced baseline renal function. These results may represent a step forward in the prediction of renal function decline in CAD patients in clinical practice. Further studies are needed to ascertain whether management strategies that consider UA and Gal-3 levels could prevent renal function decline in this population.

## Figures and Tables

**Table 1:** Baseline characteristics of the population.

Variable	Description
Age (y)	63.1 ± 12.2
Gender (Male)	411 (73.9%)
Caucasian	529 (95.1%)
Body Mass Index (kg/m^2^)	27.7 (25.4, 30.5)
Smoker	82 (14.7%)
Hypertension	362 (65.1%)
Diabetes Mellitus	126 (22.7%)
Dyslipidemia	315 (56.7%)
Previous Stroke	21 (3.8%)
Peripheral Artery Disease	15 (2.7%)
Heart Failure	60 (10.8%)
Atrial Fibrillation	35 (6.3%)
Left Ventricular Ejection Fraction < 40%	42 (7.6%)
PREVIOUS ACUTE CORONARY SYNDROME STEMI/NSTEACS	326 (58.6%)/230 (41.4%)
Complete Revascularization:	417 (75.0%)
Number of vessels diseased	
0	27 (4.9%)
1	327 (59.3%)
2	142 (25.8%)
3	55 (10.0%)
Revascularization method (%)	
0 No Revascularization	52 (9.4%)
1 Coated Stent	311 (55.9%)
2 Conventional Stent	153 (27.5%)
3 Simple angioplasty	21 (3.8%)
4 CABG	19 (3.4%)
**TREATMENT**	
Aspirin	525 (94.4%)
P2Y12 Antagonist	468 (84.2%)
Anticoagulant	29 (5.2%)
Statin	535 (96.2%)
Ezetimibe	24 (4.3%)
Fibrates	21 (3.8%)
Insulin	33 (5.9%)
Oral Antidiabetics	88 (15.8%)
ACEI	416 (74.8%)
Angiotensin Receptor Blockers	93 (16.7%)
Aldosterone Antagonist	52 (9.4%)
Betablocker	433 (77.9%)
Nitrates	45 (8.1%)
Diltiazem	11 (2.0%)
Verapamil	0 (0.0%)
Dihydropyridines	65 (11.7%)
Diuretic	130 (23.4%)
Proton Pump Inhibitors	436 (78.4%)
Digoxin	1 (0.2%)
Amiodarone	6 (1.1%)
**ANALYTICS**	
Glucose (mmol/L)	5.91 ± 1.69
Total Cholesterol (mmol/L)	3.61 ± 0.87
LDL Cholesterol (mmol/L)	1.99 ± 0.64
HDL Cholesterol (mmol/L)	1.07 ± 0.30
Non-HDL Cholesterol (mmol/L)	2.54 ± 0.75
Triglycerides (mmol/L)	1.20 ± 0.63
eGFR (mL/min/1.73 m^2^)	86.77 (72.27, 97.85)
Uric Acid (µmol/L)	341.0 ± 91.0
Hs-Tn I (ng/L)	4.0 (0.0, 11.0)
Hs-CRP (mg/L)	0.620 (0.080, 2.277)
NT-ProBNP (pmol/L)	23.6 (11.8, 49.3)
Galectin-3 (pmol/L)	337.6 ± 129.3
Phosphorus (mmol/L)	1.03 ± 0.18
Calcidiol (nmol/L)	51.1 ± 22.1
FGF23 (ng/L)	78.70 (60.50, 99.22)
Klotho (µg/L)	561.6 (465.8, 681.8)
PTH (pmol/L)	6.31 (4.88–8.05)

ACEI: angiotensin-converting enzyme inhibitors; CABG: coronary artery bypass graft; eGFR: estimated glomerular filtration rate; FGF23: fibroblast growth factor-23; HDL: high-density lipoprotein; Hs-CRP: high-sensitivity; Hs-TnI: high-sensitivity troponin I; LDL: low-density lipoprotein; NON-STEACS: non-ST elevation acute coronary syndrome; NT-proBNP: N-terminal pro-brain natriuretic peptide; PTH: parathyroid hormone; STEMI: ST-elevation myocardial infarction. Results for normally distributed quantitative variables are expressed as the mean ± standard deviation, and results for variables without a normal distribution are expressed as a median (interquartile range).

**Table 2:** Independent predictors of the percentage decrease in eGFR.

Variable	Coef.	(95% CI)	*p*	R^2^
Age (y)	0.181	(0.041, 0.321)	0.011	0.126
BMI (kg/m^2^)	0.000	(−0.000, 0.000)	0.621	
Caucasian	−3.135	(−8.763, 2.492)	0.274	
Gender (Male)	−3.072	(−5.951, −0.193)	0.037	
Hypertension	4.023	(1.335, 6.711)	0.003	
Diabetes Mellitus	0.426	(−2.797, 3.649)	0.795	
Calcidiol (nmol/L)	−0.005	(−0.009, −0.002)	0.005	
Uric Acid (µmol/L)	0.0117	(0.0031, 0.0200)	0.008	
eGFR (mL/min/1.73)	0.118	(0.030, 0.206)	0.009	
NT-ProBNP (pmol/L)	0.017	(0.000, 0.025)	0.027	
Anticoagulant	−6.350	(−11.69, −1.005)	0.020	
Insulin	5.967	(0.396, 11.54)	0.036	
Galectin-3 (nmol/L)	0.0153	(0.00088, 0.0298)	0.037	

BMI: body mass index. Other abbreviations are as for Table 1.

**Table 3:** Independent predictors of the difference in eGFR between baseline and final follow-up.

Variable	Coefficient	(95% CI)	*p*	R^2^
Age (y)	0.162	(0.056, 0.268)	0.003	0.115
BMI (kg/m^2^)	0.000	(−0.000, 0.000)	0.552	
Caucasian	−3.437	(−7.701, 0.828)	0.114	
Gender (Male)	−2.072	(−4.253, 0.109)	0.063	
Hypertension	3.420	(1.389, 5.451)	0.001	
Diabetes Mellitus	0.532	(−1.908, 2.973)	0.668	
eGFR (mL/min/1.73)	0.161	(0.095, 0.227)	<0.001	
Uric Acid (µmol/L)	0.009	(0.003, 0.017)	0.004	
Calcidiol (nmol/L)	−0.003	(−0.005, −0.001)	0.020	
Insulin	4.598	(0.388, 8.809)	0.032	
Galectin-3 (nmol/L)	0.0121	(0.0011, 0.0230)	0.031	
Anticoagulant	−4.182	(−8.217, −0.147)	0.042	

Abbreviations are as for Table 1 and Table 2.

**Table 4:** Variables independently associated with an eGFR decrease of ≥20%.

Variable	OR	(95% CI)	*p*	AUC
Age (y)	1.021	(0.996, 1.047)	0.099	0.71
BMI (kg/m^2^)	1.000	(0.999, 1.001)	0.949	
Caucasian	0.477	(0.144, 1.575)	0.224	
Gender (Male)	0.699	(0.393, 1.243)	0.222	
Hypertension	2.889	(1.387, 6.017)	0.005	
Diabetes Mellitus	1.685	(0.967, 2.937)	0.065	
Uric Acid (µmol/L)	1.237	(1.046–1.463)	0.013	
Nitrates	2.964	(1.362, 6.452)	0.006	
Anticoagulant	0.058	(0.006, 0.553)	0.013	
NT-ProBNP (pmol/L)	1.000	(1.000, 1.001)	0.049	

AUC: area under the curve. Other abbreviations are the same as for Table 1 and Table 2.

**Table 5:** Variables independently associated with absolute eGFR decrease during follow-up in patients with and without eGFR < 60 mL/min/1.73 m^2^ at baseline.

Group	Variable	Coef.	(95% CI)	*p*	R^2^
eGFR ≥ 60	Age (y)	0.182	(0.068, 0.296)	0.002	0.100
	BMI (kg/m^2^)	0.000	(−0.000, 0.000)	0.538	
	Caucasian	−4.986	(−9.408, −0.565)	0.027	
	Gender (Male)	−3.444	(−5.887, −1.001)	0.006	
	Hypertension	3.573	(1.402, 5.744)	0.001	
	Diabetes Mellitus	1.152	(−1.550, 3.853)	0.403	
	eGFR (mL/min/1.73 m^2^)	0.176	(0.083, 0.269)	<0.001	
	Uric Acid (µmol/L)	0.010	(0.004, 0.017)	0.001	
	Insulin	5.283	(0.018, 10.55)	0.049	
eGFR < 60	Age (y)	0.084	(−0.260, 0.427)	0.628	0.155
	BMI (kg/m^2^)	−0.009	(−0.524, 0.507)	0.973	
	Caucasian	8.625	(−11.42, 28.67)	0.393	
	Gender (Male)	1.208	(−3.577, 5.993)	0.615	
	Hypertension	4.601	(−1.936, 11.14)	0.164	
	Diabetes Mellitus	1.465	(−3.325, 6.254)	0.543	
	Calcidiol (nmol/L)	−0.008	(−0.015, −0.002)	0.014	

Abbreviations: As for Table 1 and Table 2.

**Table 6:** Predictor variables of major cardiovascular events during follow-up.

Predictor	HR (IC95%)	*p*	C-Stat
NT-ProBNP (pmol/L)	1.173 (1.034, 1.338)	0.014	0.77
Nitrates	2.986 (1.774, 5.024)	0.000	
Heart failure	2.668 (1.559, 4.566)	0.000	
ACEI	0.589 (0.381, 0.909)	0.017	
Proton Pump Inhibitors	3.049 (1.602, 5.802)	0.001	
BMI (kg/m^2^)	1.083 (1.033, 1.135)	0.001	
Age (y)	1.030 (1.010, 1.051)	0.004	
Statins	0.370 (0.179, 0.763)	0.007	
Dyslipidemia	1.713 (1.067, 2.752)	0.026	
PTH (pmol/L)	1.944 (1.019, 3.707)	0.043	

Abbreviations are the same as for Table 1 and Table 2.

## Data Availability

All the datasets used and/or analyzed in the study are available from the corresponding author upon reasonable request.

## Supplementary Materials

The following supporting information can be downloaded at: https://www.mdpi.com/article/10.3390/jcm14155264/s1. Table S1. Univariate Analysis. Association of variables with eGFR percentage decline during follow-up. Table S2. Independent predictors of the percentage decrease in eGFR after excluding uric acid and galectin-3 levels from the model. Table S3. Univariate Analysis. Absolute difference in the whole population between eGFR at baseline and at the end of follow-up. Table S4. Univariate Analysis. Association of variables with Individual falls of 20% or more in glomerular filtration rate during follow-up. Table S5. Univariate Analysis. Absolute differences between eGFR at baseline and at the end of follow-up according to the presence of significant CKD: Analysis stratified by glomerular filtration rate more or equal to 60. Table S6. Univariate Analysis. Absolute differences between eGFR at baseline and at the end of follow-up according to the presence of significant CKD: Analysis stratified by glomerular filtration rate less than 60. Table S7. Univariate Analysis. Association of variables as event predictors (Cox regression).

## Abbreviations

The following abbreviations are used in this manuscript:

ACEI	Angiotensin-Converting Enzyme Inhibitors
AUC	Area Under the Curve
BACS and BAMI	Biomarkers in Acute Coronary Syndrome and Biomarkers in Acute Myocardial Infarction
BMI	Body Mass Index
CABG	Coronary Artery Bypass Grafting
CI	Confidence Interval
CKD-EPI	Chronic Kidney Disease Epidemiology Collaboration equation
CKD	Chronic Kidney Disease
eGFR	Estimated Glomerular Filtration Rate
FGF-23	Fibroblast Growth Factor-23
HDL	High-Density Lipoprotein
Hs-CRP	High-Sensitivity C Reactive Protein
Hs-Tn I	High-Sensitivity Troponin I
LDL	Low-Density Lipoprotein
No-HDL	No-High-Density Lipoprotein
NSTEACS	Non-ST Elevation Acute Coronary Syndrome
NT-ProBNP	N-Terminal Pro-Brain Natriuretic Peptide
OR	Odds Ratio
PTH	Parathyroid Hormone
R2	Determination Coefficient
SCAD	Stable Coronary Artery Disease
STEMI	ST-Elevation Myocardial Infarction

## References

[1] Lv J.-C., Zhang L.-X. (2019). Prevalence and Disease Burden of Chronic Kidney Disease. Advances in Experimental Medicine and Biology.

[2] Sarnak M.J., Levey A.S., Schoolwerth A.C., Coresh J., Culleton B., Hamm L.L., McCullough P.A., Kasiske B.L., Kelepouris E., Klag M.J. (2003). Kidney disease as a risk factor for development of cardiovascular disease: A statement from the American Heart Association Councils on Kidney in Cardiovascular Disease, High Blood Pressure Research, Clinical Cardiology, and Epidemiology and Prevention. Circulation.

[3] Tonelli M., Wiebe N., Culleton B., House A., Rabbat C., Fok M., McAlister F., Garg A.X. (2006). Chronic kidney disease and mortality risk: A systematic review. J. Am. Soc. Nephrol..

[4] Couser W.G., Remuzzi G., Mendis S., Tonelli M. (2011). The contribution of chronic kidney disease to the global burden of major noncommunicable diseases. Kidney Int..

[5] Düsing P., Zietzer A., Goody P.R., Hosen M.R., Kurts C., Nickenig G., Jansen F. (2021). Vascular pathologies in chronic kidney disease: Pathophysiological mechanisms and novel therapeutic approaches. J. Mol. Med..

[6] Desmedt V., Desmedt S., Delanghe J.R., Speeckaert R., Speeckaert M.M. (2016). Galectin-3 in Renal Pathology: More Than Just an Innocent Bystander?. Am. J. Nephrol..

[7] Henderson N.C., Mackinnon A.C., Farnworth S.L., Kipari T., Haslett C., Iredale J.P., Liu F.-T., Hughes J., Sethi T. (2008). Galectin-3 expression and secretion links macrophages to the promotion of renal fibrosis. Am. J. Pathol..

[8] Alam M.L., Katz R., Bellovich K.A., Bhat Z.Y., Brosius F.C., de Boer I.H., Gadegbeku C.A., Gipson D.S., Hawkins J.J., Himmelfarb J. (2019). Soluble ST2 and Galectin-3 and Progression of CKD. Kidney Int. Rep..

[9] Tan K.C.B., Cheung C.-L., Lee A.C.H., Lam J.K.Y., Wong Y., Shiu S.W.M. (2018). Galectin-3 is independently associated with progression of nephropathy in type 2 diabetes mellitus. Diabetologia.

[10] O’Seaghdha C.M., Hwang S.J., Ho J.E., Vasan R.S., Levy D., Fox C.S. (2013). Elevated galectin-3 precedes the development of CKD. J. Am. Soc. Nephrol..

[11] Rebholz C.M., Selvin E., Liang M., Ballantyne C.M., Hoogeveen R.C., Aguilar D., McEvoy J.W., Grams M.E., Coresh J. (2018). Plasma galectin-3 levels are associated with the risk of incident chronic kidney disease. Kidney Int..

[12] Sharaf El Din U.A.A., Salem M.M., Abdulazim D.O. (2017). Uric acid in the pathogenesis of metabolic, renal, and cardiovascular diseases: A review. J. Adv. Res..

[13] Borghi C., Rosei E.A., Bardin T., Dawson J., Dominiczak A., Kielstein J.T., Manolis A.J., Perez-Ruiz F., Mancia G. (2015). Serum uric acid and the risk of cardiovascular and renal disease. J. Hypertens..

[14] Kwon Y.-E., Ahn S.-Y., Ko G.-J., Kwon Y.-J., Kim J.-E. (2024). Impact of Uric Acid Levels on Mortality and Cardiovascular Outcomes in Relation to Kidney Function. JCM.

[15] Kang D.-H., Park S.-K., Lee I.-K., Johnson R.J. (2005). Uric acid-induced C-reactive protein expression: Implication on cell proliferation and nitric oxide production of human vascular cells. J. Am. Soc. Nephrol..

[16] Mazzali M., Hughes J., Kim Y.G., Jefferson J.A., Kang D.H., Gordon K.L., Lan H.Y., Kivlighn S., Johnson R.J. (2001). Elevated uric acid increases blood pressure in the rat by a novel crystal-independent mechanism. Hypertension.

[17] Yu M.-A., Sánchez-Lozada L.G., Johnson R.J., Kang D.-H. (2010). Oxidative stress with an activation of the renin-angiotensin system in human vascular endothelial cells as a novel mechanism of uric acid-induced endothelial dysfunction. J. Hypertens..

[18] Sturm G., Kollerits B., Neyer U., Ritz E., Kronenberg F. (2008). Uric acid as a risk factor for progression of non-diabetic chronic kidney disease? The Mild to Moderate Kidney Disease (MMKD) Study. Exp. Gerontol..

[19] Madero M., Sarnak M.J., Wang X., Greene T., Beck G.J., Kusek J.W., Collins A.J., Levey A.S., Menon V. (2009). Uric Acid and Long-term Outcomes in CKD. Am. J. Kidney Dis..

[20] Nacak H., van Diepen M., Qureshi A.R., Carrero J.J., Stijnen T., Dekker F.W., Evans M. (2015). Uric acid is not associated with decline in renal function or time to renal replacement therapy initiation in a referred cohort of patients with Stage III, IV and V chronic kidney disease. Nephrol. Dial. Transplant..

[21] Liu W.-C., Hung C.-C., Chen S.-C., Yeh S.-M., Lin M.-Y., Chiu Y.-W., Kuo M.-C., Chang J.-M., Hwang S.-J., Chen H.-C. (2012). Association of hyperuricemia with renal outcomes, cardiovascular disease, and mortality. Clin. J. Am. Soc. Nephrol..

[22] Nacak H., van Diepen M., de Goeij M.C.M., Rotmans J.I., Dekker F.W., PREPARE-2 study group (2014). Uric acid: Association with rate of renal function decline and time until start of dialysis in incident pre-dialysis patients. BMC Nephrol..

[23] Chang W.X., Asakawa S., Toyoki D., Nemoto Y., Morimoto C., Tamura Y., Ota T., Shibata S., Fujigaki Y., Shen Z.Y. (2015). Predictors and the Subsequent Risk of End-Stage Renal Disease—Usefulness of 30% Decline in Estimated GFR over 2 Years. PLoS ONE.

[24] Uchida S., Chang W.X., Ota T., Tamura Y., Shiraishi T., Kumagai T., Shibata S., Fujigaki Y., Hosoyamada M., Kaneko K. (2015). Targeting Uric Acid and the Inhibition of Progression to End-Stage Renal Disease—A Propensity Score Analysis. PLoS ONE.

[25] Kumagai T., Ota T., Tamura Y., Chang W.X., Shibata S., Uchida S. (2017). Time to target uric acid to retard CKD progression. Clin. Exp. Nephrol..

[26] Kuo C.-F., Luo S.-F., See L.-C., Ko Y.-S., Chen Y.-M., Hwang J.-S., Chou I.-J., Chang H.-C., Chen H.-W., Yu K.-H. (2011). Hyperuricaemia and accelerated reduction in renal function. Scand. J. Rheumatol..

[27] Bellomo G., Venanzi S., Verdura C., Saronio P., Esposito A., Timio M. (2010). Association of uric acid with change in kidney function in healthy normotensive individuals. Am. J. Kidney Dis..

[28] Yen C.-J., Chiang C.-K., Ho L.-C., Hsu S.H.-J., Hung K.-Y., Wu K.-D., Tsai T.-J. (2009). Hyperuricemia associated with rapid renal function decline in elderly Taiwanese subjects. J. Formos. Med. Assoc..

[29] Chonchol M., Shlipak M.G., Katz R., Sarnak M.J., Newman A.B., Siscovick D.S., Kestenbaum B., Carney J.K., Fried L.F. (2007). Relationship of uric acid with progression of kidney disease. Am. J. Kidney Dis..

[30] Dawson J., Jeemon P., Hetherington L., Judd C., Hastie C., Schulz C., Sloan W., Muir S., Jardine A., McInnes G. (2013). Serum uric acid level, longitudinal blood pressure, renal function, and long-term mortality in treated hypertensive patients. Hypertension.

[31] Iseki K., Oshiro S., Tozawa M., Iseki C., Ikemiya Y., Takishita S. (2001). Significance of hyperuricemia on the early detection of renal failure in a cohort of screened subjects. Hypertens. Res..

[32] Ficociello L.H., Rosolowsky E.T., Niewczas M.A., Maselli N.J., Weinberg J.M., Aschengrau A., Eckfeldt J.H., Stanton R.C., Galecki A.T., Doria A. (2010). High-normal serum uric acid increases risk of early progressive renal function loss in type 1 diabetes: Results of a 6-year follow-up. Diabetes Care.

[33] Altemtam N., Russell J., El Nahas M. (2012). A study of the natural history of diabetic kidney disease (DKD). Nephrol. Dial. Transplant..

[34] Kendrick J., Cheung A.K., Kaufman J.S., Greene T., Roberts W.L., Smits G., Chonchol M. (2012). Associations of plasma 25-hydroxyvitamin D and 1,25-dihydroxyvitamin D concentrations with death and progression to maintenance dialysis in patients with advanced kidney disease. Am. J. Kidney Dis..

[35] Balaguer J., García-Foncillas J., Tuñón J. (2024). Natriuretic peptides: Another tool for the management of cancer?. Crit. Rev. Oncol. Hematol..

[36] Tuñón J., Blanco-Colio L., Cristóbal C., Tarín N., Higueras J., Huelmos A., Alonso J., Egido J., Asensio D., Lorenzo Ó. (2014). Usefulness of a combination of monocyte chemoattractant protein-1, galectin-3, and N-terminal probrain natriuretic peptide to predict cardiovascular events in patients with coronary artery disease. Am. J. Cardiol..

[37] Cohen J., Cohen J. (1988). Multiple regression and correlation analysis. Statistical Power Analysis for the Behavioral Sciences.

[38] Kim A.J., Ro H., Kim H., Chang J.H., Lee H.H., Chung W., Jung J.Y. (2021). Soluble ST2 and Galectin-3 as Predictors of Chronic Kidney Disease Progression and Outcomes. Am. J. Nephrol..

[39] Chen S.C., Kuo P.L. (2016). The role of galectin-3 in the kidneys. Int. J. Mol. Sci..

[40] Chade A.R., Lerman A., Lerman L.O. (2005). Kidney in early atherosclerosis. Hypertension.

[41] Lau E.S., Liu E., Paniagua S.M., Sarma A.A., Zampierollo G., López B., Díez J., Wang T.J., Ho J.E. (2021). Galectin-3 Inhibition with Modified Citrus Pectin in Hypertension. JACC Basic Transl. Sci..

[42] Ohno I., Hosoya T., Gomi H., Ichida K., Okabe H., Hikita M. (2001). Serum uric acid and renal prognosis in patients with IgA nephropathy. Nephron.

[43] Tsai C.W., Lin S.Y., Kuo C.C., Huang C.C. (2017). Serum Uric Acid and Progression of Kidney Disease: A Longitudinal Analysis and Mini-Review. PLoS ONE.

[44] Waas T., Schulz A., Lotz J., Rossmann H., Pfeiffer N., Beutel M.E., Schmidtmann I., Münzel T., Wild P.S., Lackner K.J. (2021). Distribution of estimated glomerular filtration rate and determinants of its age dependent loss in a German population-based study. Sci. Rep..

[45] Kuźma Ł., Kulikowska A., Kurasz A., Niwińska M.M., Zalewska-Adamiec M., Dobrzycki S., Bachórzewska-Gajewska H. (2020). The effect of serum uric acid levels on the long-term prognosis of patients with non-ST-elevation myocardial infarction. Adv. Clin. Exp. Med..

[46] Neri L., Rocca Rey L.A., Lentine K.L., Hinyard L.J., Pinsky B., Xiao H., Dukes J., Schnitzler M.A. (2011). Joint association of hyperuricemia and reduced GFR on cardiovascular morbidity: A historical cohort study based on laboratory and claims data from a national insurance provider. Am. J. Kidney Dis..

[47] Niskanen L.K., Laaksonen D.E., Nyyssönen K., Alfthan G., Lakka H.-M., Lakka T.A., Salonen J.T. (2004). Uric acid level as a risk factor for cardiovascular and all-cause mortality in middle-aged men: A prospective cohort study. Arch. Intern. Med..

[48] Wang R., Song Y., Yan Y., Ding Z. (2016). Elevated serum uric acid and risk of cardiovascular or all-cause mortality in people with suspected or definite coronary artery disease: A meta-analysis. Atherosclerosis.

[49] Jansen H., Koenig W., Jaensch A., Mons U., Breitling L.P., Scharnagl H., Stojakovic T., Schunkert H., Brenner H., Rothenbacher D. (2016). Prognostic Utility of Galectin-3 for Recurrent Cardiovascular Events During Long-term Follow-up in Patients with Stable Coronary Heart Disease: Results of the KAROLA Study. Clin. Chem..

[50] Daniels L.B., Maisel A.S. (2007). Natriuretic peptides. J. Am. Coll. Cardiol..

[51] Bansal N., Zelnick L., Robinson-Cohen C., Hoofnagle A.N., Ix J.H., Lima J.A., Shoben A.B., Peralta C.A., Siscovick D.S., Kestenbaum B. (2014). Serum Parathyroid Hormone and 25-Hydroxyvitamin D Concentrations and Risk of Incident Heart Failure: The Multi-Ethnic Study of Atherosclerosis. J. Am. Heart Assoc..

[52] Wannamethee S.G., Welsh P., Papacosta O., Lennon L., Whincup P.H., Sattar N. (2014). Elevated Parathyroid Hormone, But Not Vitamin D Deficiency, Is Associated with Increased Risk of Heart Failure in Older Men with and Without Cardiovascular Disease. Circ. Heart Fail..

[53] Schlüter K.D., Piper H.M. (1992). Trophic effects of catecholamines and parathyroid hormone on adult ventricular cardiomyocytes. Am. J. Physiol..

[54] Aceña A., Pello A.M., Carda R., Lorenzo O., Gonzalez-Casaus M.L., Blanco-Colio L.M., Martín-Ventura J.L., Palfy J., Orejas M., Rábago R. (2015). Parathormone levels are independently associated with the presence of left ventricular hypertrophy in patients with coronary artery disease. J. Nutr. Health Aging.

[55] Meems L.M.G., Brouwers F.P., Joosten M.M., Lambers Heerspink H.J., de Zeeuw D., Bakker S.J.L., Gansevoort R.T., van Gilst W.H., van der Harst P., de Boer R.A. (2016). Plasma calcidiol, calcitriol, and parathyroid hormone and risk of new onset heart failure in a population-based cohort study. ESC Heart Fail..

[56] Folsom A.R., Alonso A., Misialek J.R., Michos E.D., Selvin E., Eckfeldt J.H., Coresh J., Pankow J.S., Lutsey P.L. (2014). Parathyroid hormone concentration and risk of cardiovascular diseases: The Atherosclerosis Risk in Communities (ARIC) Study. Am. Heart. J..

[57] Kamycheva E., Sundsfjord J., Jorde R. (2004). Serum parathyroid hormone levels predict coronary heart disease: The Tromsø Study. Eur. J. Cardiovasc. Prev. Rehabil..

[58] Malluche H.H., Blomquist G., Monier-Faugere M.-C., Cantor T.L., Davenport D.L. (2015). High Parathyroid Hormone Level and Osteoporosis Predict Progression of Coronary Artery Calcification in Patients on Dialysis. J. Am. Soc. Nephrol..

[59] Pilz S., Tomaschitz A., Drechsler C., Ritz E., Boehm B.O., Grammer T.B., März W. (2010). Parathyroid hormone level is associated with mortality and cardiovascular events in patients undergoing coronary angiography. Eur. Heart J..

[60] Yang B., Lu C., Wu Q., Zhang J., Zhao H., Cao Y. (2016). Parathyroid hormone, cardiovascular and all-cause mortality: A meta-analysis. Clin. Chim. Acta.

[61] Palmer S.C., Hayen A., Macaskill P., Pellegrini F., Craig J.C., Elder G.J., Strippoli G.F.M. (2011). Serum Levels of Phosphorus, Parathyroid Hormone, and Calcium and Risks of Death and Cardiovascular Disease in Individuals with Chronic Kidney Disease. JAMA.

