# Supplementary material for: High Levels of Galectin-3 and Uric Acid Are Independent Predictors of Renal Impairment in Patients with Stable Coronary Artery Disease
Source: J Clin Med. 2025 Jul 25;14(15):5264. doi: 10.3390/jcm14155264 (PMC12347710; doi:10.3390/jcm14155264)
Supplement: Supplementary file 1 [file jcm-14-05264-s001.zip › jcm-3687326-supplementary.pdf]

SUPPLEMENTARY INFORMATION

High levels of galectin-3 and uric acid are independent predictors of renal impairment in patients with stable coronary artery disease

Nayleth Leal-Pérez [1]; Luis M. Blanco-Colio [2,3]; José Luis Martín-Ventura [2,3,4]; Carlos Gutiérrez-Landaluce [5]; Ignacio Mahíllo-Fernández [6]; María Luisa González-Casaus [7]; Óscar Lorenzo [4,8,9], Jesús Egido [2,4,8,9,+], José Tuñón [2,3,4,10,+,*].

1. Department of Gynecology and Obstetrics, Príncipe de Asturias University Hospital, Madrid, 28805, Spain
2. Laboratory of Vascular Pathology, IIS-Fundación Jiménez Díaz, Madrid, 28040, Spain
3. CIBERCV, Madrid, 28029, Spain
4. Autónoma University, Madrid, 28049, Spain
5. Department of Cardiology, Hospital Universitario de Fuenlabrada, Madrid, 28942, Spain
6. Research Unit, IIS-Fundación Jiménez Díaz, Madrid, 28040, Spain
7. Laboratory of Nephrology and Mineral Metabolism, Hospital La Paz, Madrid, 28046, Spain
8. Renal,Vascular and Diabetes research Laboratory, IIS-Fundación Jiménez Díaz, Madrid, 28040, Spain
9. Diabetes and Associated Metabolic Diseases Networking (CIBERDEM), Madrid, 28029, Spain
10. Department of Cardiology, IIS-Fundación Jiménez Díaz, Madrid, 28040, Spain

[+] Both senior authors contributed equally to this paper

[*]CORRESPONDING AUTHOR:
José Tuñón, MD, PHD
Department of Cardiology, IIS-Fundación Jiménez Díaz,
Avenida Reyes Católicos 2, 28040 Madrid (Spain), Phone 00-34-915504800, Ext 3701. Email: jtunon@fjd.es

Table S1. Univariate Analysis. Association of variables with eGFR percentage decline during follow-up

Variable	Coefficient	(95% CI)	P
Age (y)	0.177	(0.077, 0.276)	0.001
BMI	0.000	(-0.000, 0.000)	0.887
Caucasian	-2.131	(-7.813, 3.551)	0.462
Gender (Male)	-3.651	(-6.431, -0.872)	0.010
Smoker	-2.082	(-5.562, 1.399)	0.241
Hypertension	6.189	(3.655, 8.722)	<0.001
Diabetes Mellitus	3.597	(0.680, 6.514)	0.016
Dyslipidemia	0.913	(-1.568, 3.395)	0.470
Previous Stroke	0.445	(-5.964, 6.854)	0.892
Peripheral artery disease	0.717	(-6.824, 8.257)	0.852
Heart failure	0.443	(-3.527, 4.412)	0.827
Atrial fibrillation	-1.458	(-6.557, 3.641)	0.575
Left ventricular ejection fraction <40%	1.946	(-2.687, 6.580)	0.410
ST-elevation Myocardial Infarction (STEMI)	-2.024	(-4.515, 0.466)	0.111
Number of affected vessels in the last event	1.723	(0.062, 3.383)	0.042
Complete revascularization	-1.045	(-3.879, 1.789)	0.469
Revascularization method (%)			0.256
No revascularization	Ref.		
Coated stent	2.982	(-1.327, 7.291)	0.175
Conventional stent	2.873	(-1.760, 7.506)	0.224
Simple angioplasty	-2.856	(-10.42, 4.706)	0.458
CABG	-0.454	(-8.158, 7.251)	0.908
**ANALYTICS**			
Glucose (mmol/L)	0.00139	(-0.00083, 0.00361)	0.217
Total cholesterol (mmol/L)	0.00088	(-0.00005, 0.00181)	0.067
LDL cholesterol (mmol/L)	0.00072	(-0.00054, 0.00202)	0.264
HDL cholesterol (mmol/L)	0.00140	(-0.00142, 0.00419)	0.330
Non-HDL cholesterol (mmol/L)	0.00101	(-0.00008, 0.00212)	0.067
Triglycerides (mmol/L)	0.00028	(0.00002, 0.00053)	0.030
Uric acid (µmol/L)	57.56	(9.64, 105.36)	0.019
eGFR (mL/min/1.73m^2)	-0.063	(-0.124, -0.002)	0.044
Hs-Tn I (ng/mL)	-0.631	(-5.465, 4.203)	0.798
Hs-CRP (mg/L)	0.066	(-0.058, 0.189)	0.296
NT-ProBNP (pmol/L)	0.017	(0.009, 0.047)	<0.001
Galectin-3 (pmol/L)	63.5	(27.4, 99.6)	0.001
Phosphorus (mmol/L)	0.00252	(-0.0692, 0.0742)	0.945
Calcidiol (nmol/L)	-0.658	(-1.000, -0.312)	<0.001
FGF23 (pg/mL)	-0.002	(-0.012, 0.007)	0.601
Klotho (ng/mL)	-0.003	(-0.008, 0.002)	0.275
PTH (pmol/L)	0.0035	(-0.00095, 0.00807)	0.124

**TREATMENT**			
Aspirin	-1.090	(-6.415, 4.235)	0.688
P2Y12 antagonist	-0.930	(-4.328, 2.468)	0.591
Anticoagulant	-4.122	(-9.61, 1.363)	0.140
Statin	2.116	(-4.291, 8.523)	0.517
Ezetimibe	-3.916	(-9.92, 2.088)	0.201
Fibrates	1.562	(-4.998, 8.122)	0.640
Insulin	7.473	(2.339, 12.61)	0.004
Oral antidiabetics	3.220	(-0.137, 6.576)	0.060
ACEI	-1.817	(-4.656, 1.021)	0.209
Angiotensin receptor blockers	2.752	(-0.533, 6.036)	0.100
Aldosterone antagonist	3.216	(-1.011, 7.443)	0.136
Betablocker	0.669	(-2.280, 3.619)	0.656
Nitrates	5.378	(0.919, 9.84)	0.018
Diltiazem	9.083	(0.343, 17.82)	0.042
Dihydropyridines	4.633	(0.847, 8.419)	0.017
Diuretic	4.354	(1.476, 7.232)	0.003
Proton Pump Inhibitors	1.105	(-1.897, 4.107)	0.470
Amiodarone	-4.674	(-16.49, 7.142)	0.437

Abbreviations: ACEI: Angiotensin-converting enzyme inhibitors, ARB: Angiotensin receptor blockers, BMI: Body Mass Index, CABG: Coronary Artery Bypass Grafting. CKD-EPI: Chronic Kidney Disease Epidemiology Collaboration equation. eGFR: Estimated Glomerular Filtration Rate. FGF-23: Fibroblast Growth Factor 23. HDL: High-density lipoprotein. Hs-CRP: High- Sensitivity C Reactive Protein. Hs-Tn I: High-Sensitivity Troponin I. LDL: low- density lipoprotein. No-HDL: No-High-density lipoprotein. NT-ProBNP: N- Terminal pro-Brain Natriuretic Peptide. PTH: Parathyroid hormone. STEMI: ST- elevation Myocardial Infarction.

Table S2. Independent predictors of the percentage decrease in eGFR after excluding uric acid and galectin-3 levels from the model

**Variable**	**Coef.**	**(95% CI)**	**P**
Age (y)	0.161	(0.025, 0.297)	0.020
BMI (kg/m^2)	0.000	(-0.000, 0.000)	0.660
Caucasian	-3.614	(-9.268, 2.040)	0.210
Gender (Male)	-2.411	(-5.147, 0.325)	0.084
Hypertension	4.267	(1.569, 6.964)	0.002
Diabetes Mellitus	0.782	(-2.448, 4.012)	0.634
Calcidiol (nmol/L)	-0.503	(-0.856, -0.150)	0.005
eGFR (mL/min/1.73m^2)	0.070	(-0.013, 0.154)	0.099
NT-ProBNP (pmol/L)	0.034	(0.000, 0.051)	0.011
Anticoagulant	-6.344	(-11.71, -0.982)	0.020
Insulin	5.543	(-0.077, 11.16)	0.053

$R^2 = 0.105$

Table S3. Univariate Analysis. Absolute difference in the whole population between eGFR at baseline and at the end of follow-up

Variable	Coefficient	(95% CI)	P
Age (y)	0.049	(-0.027, 0.125)	0.203
BMI	0.000	(-0.000, 0.000)	0.794
Caucasian	-3.716	(-7.990, 0.557)	0.088
Gender (Male)	-1.800	(-3.902, 0.302)	0.093
Smoker	-0.484	(-3.110, 2.142)	0.718
Hypertension	3.846	(1.924, 5.769)	<0.001
Diabetes Mellitus	2.711	(0.512, 4.909)	0.016
Dyslipidemia	0.910	(-0.959, 2.780)	0.339
Stroke	-1.729	(-6.557, 3.100)	0.482
Peripheral artery disease	1.429	(-4.253, 7.112)	0.621
Heart failure	-1.108	(-4.098, 1.883)	0.467
Atrial fibrillation	-2.090	(-5.930, 1.750)	0.286
Left ventricular ejection fraction <40%	0.255	(-3.239, 3.750)	0.886
ST-elevation Myocardial Infarction (STEMI)	-1.258	(-3.137, 0.620)	0.189
Number of affected vessels in the last event	1.187	(-0.065, 2.439)	0.063
Complete revascularization	-0.081	(-2.218, 2.056)	0.941
Revascularization method (%)			0.211
No revascularization	Ref.		
Coated stent	1.957	(-1.289, 5.203)	0.237
Conventional stent	1.452	(-2.038, 4.943)	0.414
Simple angioplasty	-3.393	(-9.090, 2.304)	0.243
CABG	-0.320	(-6.124, 5.485)	0.914
**ANALYTICS**			
Glucose (mmol/L)	0.216	(-0.324, 0.755)	0.432
Total cholesterol (mmol/L)	0.492	(-0.233, 1.193)	0.178
LDL cholesterol (mmol/L)	0.362	(-0.620, 1.319)	0.477
HDL cholesterol (mmol/L)	0.466	(-1.656, 2.588)	0.664
Non-HDL cholesterol (mmol/L)	0.619	(-0.206, 1.446)	0.137
Triglycerides (mmol/L)	0.215	(0.023, 0.407)	0.030
Uric Acid (µmol/L)	0.0081	(-0.0021, 0.0181)	0.119
eGFR (mL/min/1.73m²)	0.033	(-0.014, 0.079)	0.166
Hs-Tn I (ng/L)	-0.649	(-4.292, 2.995)	0.727
Hs-CRP (mg/L)	0.031	(-0.061, 0.123)	0.512
NT-ProBNP (pmol/L)	0.00012	(-0.00005, 0.00029)	0.276
Galectin-3 (pmol/L)	0.014	(0.003, 0.025)	0.011
Phosphorus (mmol/L)	-1.355	(-55.41, 52.70)	0.960
Calcidiol (nmol/L)	-0.414	(-0.674, -0.155)	0.002
FGF23 (pmol/L)	-0.00022	(-0.00061, 0.00017)	0.292

Klotho (pmol/L)	-0.015	(-0.076, 0.046)	0.637
PTH (pmol/L)	0.00063	(-0.0027, 0.0039)	0.700
**TREATMENT**			
Aspirin	-0.087	(-4.101, 3.928)	0.966
P2Y12 antagonist	-0.725	(-3.286, 1.837)	0.579
Anticoagulant	-3.954	(-8.083, 0.175)	0.061
Statin	2.959	(-1.866, 7.783)	0.229
Ezetimibe	-3.053	(-7.577, 1.471)	0.186
Fibrates	1.780	(-3.163, 6.723)	0.480
Insulin	4.946	(1.070, 8.822)	0.012
Oral antidiabetics	2.693	(0.165, 5.220)	0.037
ACEI	-1.136	(-3.276, 1.004)	0.298
Angiotensin receptor blockers	1.855	(-0.621, 4.332)	0.142
Aldosterone antagonist	1.077	(-2.114, 4.268)	0.508
Betablocker	0.822	(-1.400, 3.044)	0.468
Nitrates	2.594	(-0.777, 5.965)	0.131
Diltiazem	3.238	(-3.369, 9.840)	0.336
Dihydropyridines	2.700	(-0.159, 5.559)	0.064
Diuretic	2.245	(0.067, 4.424)	0.043
Proton Pump Inhibitors	1.140	(-1.121, 3.402)	0.322
Amiodarone	-3.503	(-12.41, 5.404)	0.440

Abbreviations as for table S1.

Table S4. Univariate Analysis. Association of variables with Individual falls of 20% or more in glomerular filtration rate during follow-up

**Variable**	**OR**	**(95% CI)**	**P**
Age (y)	1.038	(1.017, 1.059)	<0.001
BMI	1.000	(0.999, 1.001)	0.934
Caucasian	1.015	(0.341, 3.022)	0.979
Gender (Male)	0.600	(0.364, 0.990)	0.046
Smoker	0.784	(0.385, 1.594)	0.501
Hypertension	4.547	(2.284, 9.055)	<0.001
Diabetes Mellitus	1.967	(1.182, 3.274)	0.009
Dyslipidemia	1.181	(0.732, 1.906)	0.495
Previous Stroke	0.587	(0.134, 2.577)	0.479
Peripheral artery disease	0.398	(0.051, 3.080)	0.377
Heart failure	1.346	(0.666, 2.720)	0.407
Atrial fibrillation	0.976	(0.366, 2.606)	0.962
Left ventricular ejection fraction <40%	2.184	(1.047, 4.555)	0.037
ST-elevation Myocardial Infarction (STEMI)	0.605	(0.377, 0.970)	0.037
Number of affected vessels in the last event	1.366	(1.006, 1.855)	0.045
Complete revascularization	0.683	(0.409, 1.141)	0.145
Revascularization method (%)			0.491

No revascularization	Ref.		
Coated stent	1.778	(0.672, 4.707)	0.246
Conventional stent	1.911	(0.689, 5.296)	0.213
Simple angioplasty	0.495	(0.054, 4.542)	0.533
CABG	1.106	(0.195, 6.269)	0.909
**ANALYTICS**			
Glucose (mmol/L)	1.073	(0.983, 1.172)	0.304
Total cholesterol (mmol/L)	1.162	(1.002, 1.224)	0.087
LDL cholesterol (mmol/L)	1.134	(0.931, 1.288)	0.314
HDL cholesterol (mmol/L)	1.187	(0.735, 1.343)	0.520
Non-HDL cholesterol (mmol/L)	1.186	(1.002, 1.257)	0.072
Triglycerides (mmol/L)	1.061	(1.017, 1.106)	0.019
Uric acid (μmol/L)	1.014	(1.009, 1.020)	0.001
eGFR (mL/min/1.73m^2)	0.977	(0.967, 0.989)	<0.001
Hs-Tn I (ng/L)	0.764	(0.137, 4.262)	0.758
Hs-CRP (mg/L)	0.996	(0.970, 1.021)	0.735
NT-ProBNP (pmol/L)	1.007	(1.006, 1.009)	<0.001
Galectin-3 (pmol/L)	41.7	(39.1, 44.5)	0.015
Phosphorus (mmol/L)	0.751	(0.488, 1.153)	0.434
Calcidiol (nmol/L)	0.922	(0.876, 0.968)	0.011
FGF23 (ng/L)	1.000	(0.999, 1.002)	0.665
Klotho (μg/L)	1.000	(0.999, 1.001)	0.688
PTH (pmol/L)	1.009	(1.001, 1.017)	0.016
**TREATMENT**			
Aspirin	0.912	(0.339, 2.453)	0.855
P2Y12 antagonist	0.972	(0.510, 1.854)	0.932
Anticoagulant	0.193	(0.026, 1.441)	0.108
Statin	1.060	(0.304, 3.695)	0.926
Ezetimibe	0.803	(0.233, 2.763)	0.727
Fibrates	1.439	(0.468, 4.429)	0.525
Insulin	3.120	(1.448, 6.722)	0.004
Oral antidiabetics	1.916	(1.086, 3.381)	0.025
ACEI	0.590	(0.356, 0.979)	0.041
Angiotensin receptor blockers	1.761	(1.001, 3.096)	0.049
Aldosterone antagonist	1.436	(0.688, 3.000)	0.335
Betablocker	1.037	(0.588, 1.829)	0.900
Nitrates	3.246	(1.657, 6.360)	0.001
Diltiazem	2.169	(0.562, 8.378)	0.261
Dihydropyridines	2.058	(1.104, 3.836)	0.023
Diuretic	2.133	(1.291, 3.525)	0.003
Proton Pump Inhibitors	0.817	(0.470, 1.420)	0.473
Amiodarone	0.000	(0.000, Inf)	0.981

Abbreviations as for table S1.

Table S5. Univariate Analysis. Absolute differences between eGFR at baseline and at the end of follow-up according to the presence of significant CKD: Analysis stratified by glomerular filtration rate more or equal to 60.

Variable	Coefficient	(95% CI)	P
Age (y)	0.063	(-0.023, 0.150)	0.149
BMI	0.000	(-0.000, 0.000)	0.757
Caucasian	-3.994	(-8.458, 0.470)	0.079
Gender (Male)	-2.063	(-4.433, 0.307)	0.088
Smoker	-0.867	(-3.659, 1.925)	0.542
Hypertension	4.076	(2.013, 6.140)	<0.001
Diabetes Mellitus	3.252	(0.762, 5.742)	0.011
Dyslipidemia	0.905	(-1.145, 2.955)	0.386
Previous Stroke	-4.240	(-10.72, 2.240)	0.199
Peripheral artery disease	4.055	(-2.708, 10.82)	0.239
Heart failure	-1.795	(-5.460, 1.869)	0.336
Atrial fibrillation	-2.386	(-6.859, 2.087)	0.295
Left ventricular ejection fraction <40%	-0.715	(-4.969, 3.539)	0.741
ST-elevation Myocardial Infarction (STEMI)	-1.242	(-3.305, 0.821)	0.238
Number of affected vessels in the last event	1.435	(0.030, 2.839)	0.045
Complete revascularization	-0.080	(-2.502, 2.342)	0.948
Revascularization method (%)			0.247
No revascularization	Ref.		
Coated stent	1.184	(-2.380, 4.748)	0.514
Conventional stent	0.662	(-3.184, 4.508)	0.735
Simple angioplasty	-5.067	(-11.24, 1.109)	0.108
CABG	0.017	(-6.586, 6.620)	0.996
**ANALYTICS**			
Glucose (mmol/L)	0.305	(-0.426, 1.035)	0.423
Total cholesterol (mmol/L)	0.811	(-0.425, 2.013)	0.195
LDL cholesterol (mmol/L)	0.656	(-1.005, 2.316)	0.437
HDL cholesterol (mmol/L)	0.347	(-3.123, 3.817)	0.844
Non-HDL cholesterol (mmol/L)	1.081	(-0.309, 2.474)	0.129
Triglycerides (mmol/L)	1.681	(0.088, 3.273)	0.044
Uric acid (μmol/L)	0.011	(-0.798, 1.384)	0.068
eGFR (mL/min/1.73m²)	0.043	(-0.028, 0.114)	0.233
Hs-Tn I (ng/L)	-0.744	(-4.472, 2.984)	0.695
Hs-CRP (mg/L)	0.039	(-0.089, 0.168)	0.546
NT-ProBNP (pmol/L)	0.009	(-0.009, 0.027)	0.282
Galectin-3 (pmol/L)	18.3	(6.2, 30.7)	0.003
Phosphorus (mmol/L)	0.542	(-5.305, 6.388)	0.855
Calcidiol (nmol/L)	-0.355	(-0.641, -0.068)	0.015
FGF23 (ng/L)	-0.004	(-0.012, 0.005)	0.402
Klotho (μg/L)	-0.002	(-0.006, 0.002)	0.358
PTH (pmol/L)	0.870	(-1.780, 5.610)	0.308

**TREATMENT**			
Aspirin	-0.659	(-5.310, 3.991)	0.781
P2Y12 antagonist	-1.953	(-4.893, 0.986)	0.192
Anticoagulant	-4.689	(-9.74, 0.366)	0.069
Statin	4.353	(-1.458, 10.17)	0.142
Ezetimibe	-3.612	(-8.347, 1.122)	0.134
Fibrates	2.712	(-2.618, 8.041)	0.318
Insulin	6.394	(1.472, 11.32)	0.011
Oral antidiabetics	3.449	(0.592, 6.305)	0.018
ACEI	-1.535	(-3.916, 0.845)	0.206
Angiotensin receptor blockers	2.063	(-0.666, 4.791)	0.138
Aldosterone antagonist	0.689	(-3.066, 4.443)	0.719
Betablocker	1.312	(-1.123, 3.747)	0.290
Nitrates	3.301	(-0.876, 7.478)	0.121
Diltiazem	1.763	(-6.152, 9.680)	0.662
Dihydropyridines	3.375	(0.009, 6.741)	0.049
Diuretic	2.623	(0.118, 5.129)	0.040
Proton Pump Inhibitors	1.743	(-0.769, 4.255)	0.173
Amiodarone	-2.876	(-14.02, 8.269)	0.612

Abbreviations as for table S1.

Table S6. Univariate Analysis. Absolute differences between eGFR at baseline and at the end of follow-up according to the presence of significant CKD: Analysis stratified by glomerular filtration rate less than 60.

Variable	Coef.	(95% CI)	P
Age (y)	0.204	(-0.089, 0.498)	0.169
BMI	0.003	(-0.514, 0.519)	0.992
Caucasian	5.619	(-13.03, 24.27)	0.550
Gender (Male)	-1.306	(-5.777, 3.165)	0.562
Smoker	3.404	(-5.168, 11.98)	0.431
Hypertension	4.703	(-1.822, 11.23)	0.155
Diabetes Mellitus	1.041	(-3.526, 5.607)	0.651
Dyslipidemia	0.945	(-3.492, 5.381)	0.672
Previous Stroke	2.748	(-3.842, 9.337)	0.408
Peripheral artery disease	-5.420	(-14.89, 4.046)	0.257
Heart failure	1.435	(-3.530, 6.400)	0.566
Atrial fibrillation	-0.570	(-7.537, 6.397)	0.871
Left ventricular ejection fraction <40%	3.574	(-2.111, 9.258)	0.214
ST-elevation Myocardial Infarction (STEMI)	-1.516	(-5.929, 2.897)	0.495
Number of affected vessels in the last event	0.366	(-2.354, 3.087)	0.789
Complete revascularization	-0.805	(-5.283, 3.673)	0.721
Revascularization method (%)			0.299
No revascularization	Ref.		
Coated stent	6.846	(-0.721, 14.41)	0.075

Conventional stent	6.571	(-1.378, 14.52)	0.104
Simple angioplasty	9.510	(-5.177, 24.20)	0.200
CABG	0.673	(-10.81, 12.15)	0.907
**ANALYTICS**			
Glucose (mmol/L)	0.198	(-0.535, 0.934)	0.599
Total cholesterol (mmol/L)	0.656	(-1.468, 2.781)	0.550
LDL cholesterol (mmol/L)	0.193	(-2.548, 2.983)	0.881
HDL cholesterol (mmol/L)	3.473	(-4.208, 11.104)	0.370
Non-HDL cholesterol (mmol/L)	0.541	(-1.932, 3.001)	0.673
Triglycerides (mmol/L)	1.681	(-1.858, 5.227)	0.343
Uric acid (µmol/L)	0.003	(-18.276, 24.323)	0.777
eGFR (mL/min/1.73m^2)	0.003	(-0.219, 0.225)	0.979
Hs-Tn I (ng/L)	103.100	(-3.850, 210.1)	0.059
Hs-CRP (mg/L)	0.036	(-0.084, 0.155)	0.553
NT-ProBNP (pmol/L)	0.009	(-0.009, 0.018)	0.238
Galectin-3 (pmol/L)	4.73	(−17.96, 27.45)	0.678
Phosphorus (mmol/L)	-2.411	(-13.144, 8.682)	0.655
Calcidiol (nmol/L)	-0.818	(-1.390, -0.247)	0.006
FGF23 (ng/L)	-0.003	(-0.014, 0.009)	0.612
Klotho (µg/L)	0.013	(-0.002, 0.028)	0.098
PTH (pmol/L)	-0.950	(-0.366, 1.760)	0.478
**TREATMENT**			
Aspirin	1.460	(-5.923, 8.842)	0.694
P2Y12 antagonist	3.611	(-1.290, 8.512)	0.146
Anticoagulant	-1.651	(-8.262, 4.959)	0.620
Statin	-1.293	(-9.208, 6.622)	0.745
Ezetimibe	6.777	(-11.85, 25.40)	0.470
Fibrates	-6.852	(-20.07, 6.362)	0.304
Insulin	3.362	(-2.461, 9.185)	0.253
Oral antidiabetics	-0.202	(-5.365, 4.960)	0.938
ACEI	0.691	(-4.014, 5.396)	0.770
Angiotensin receptor blockers	0.682	(-5.013, 6.377)	0.812
Aldosterone antagonist	3.083	(-2.565, 8.732)	0.280
Betablocker	-2.462	(-7.702, 2.778)	0.352
Nitrates	2.064	(-3.311, 7.438)	0.446
Diltiazem	7.922	(-2.864, 18.71)	0.147
Dihydropyridines	1.424	(-3.728, 6.576)	0.583
Diuretic	1.883	(-2.536, 6.302)	0.398
Proton Pump Inhibitors	-2.513	(-7.453, 2.427)	0.314
Amiodarone	-4.132	(-17.41, 9.147)	0.537

Abbreviations as for table S1.

Table S7. Univariate Analysis. Association of variables as event predictors (Cox regression)

Variable	HR (IC95%)	P - value
Age	1.048 (1.029, 1.067)	<0.001
Gender (male)	0.799 (0.515, 1.238)	0.314
Black race		
Smoker	0.429 (0.187, 0.982)	0.045
Hypertension	2.766 (1.564, 4.891)	<0.001
Diabetes Mellitus	1.499 (0.956, 2.351)	0.078
BMI	1.048 (1.007, 1.091)	0.022
Dyslipidemia	1.394 (0.910, 2.133)	0.127
Stroke	2.684 (1.345, 5.353)	0.005
Peripheral vascular disease	0.915 (0.225, 3.720)	0.901
Heart failure	3.148 (1.944, 5.097)	<0.001
Atrial fibrillation	2.085 (1.109, 3.919)	0.022
Ejection fraction < 40%	1.706 (0.929, 3.134)	0.085
**STEMI**	0.689 (0.453, 1.049)	0.082
**Number of vessel disease**		
1	1.232 (0.441, 3.443)	0.691
2	1.933 (0.677, 5.519)	0.218
3	2.071 (0.681, 6.297)	0.199
**Revascularization method**		
Coated Stent	0.818 (0.441, 1.518)	0.524
Conventional Stent	0.839 (0.430, 1.638)	0.607
Simple angioplasty	0.781 (0.221, 2.754)	0.700
CABG	0.529 (0.119, 2.354)	0.404
Complete revascularization	0.504 (0.332, 0.765)	0.001
**ANALYTICS**		
Glucose (mmol/L)	1.616 (0.599, 4.350)	0.347
Cholesterol (mmol/L)	1.993 (0.206, 18.921)	0.557
LDL (mmol/L)	1.080 (0.047, 24.296)	0.960
Non-HDL (mmol/L)	2.698 (0.215, 34.632)	0.446
Triglycerides (mmol/L)	11.541 (0.641, 189.501)	0.093

HDL (mmol/L)	1.260 (0.001, 1081.248)	0.946
Creatinine (μmol/L)	1.015 (1.009, 1.021)	<0.001
eGFR (mL/min/1.73m²)	0.770 (0.701, 0.846)	<0.001
Uric acid (μmol/L)	1.002 (1.000, 1.005)	0.031
Hypersensitive troponin (ng/L)	1.000 (0.999, 1.001)	0.928
HS C-reactive protein (mg/L)	1.019 (0.861, 1.206)	0.827
NT-ProBNP (pmol/L)	1.263 (1.163, 1.371)	<0.001
Galectin-3 (μg/L)	10.157 (2.178, 46.580)	0.003
Phosphorus (mmol/L)	1.742 (0.555, 5.450)	0.341
Calcidiol (nmol/L)	0.886 (0.798, 0.983)	0.023
FGF23 (ng/L)	1.162 (1.060, 1.275)	0.001
Klotho (μg/L)	0.631 (0.244, 1.633)	0.343
PTH (pmol/L)	2.912 (1.795, 4.725)	<0.001
**TREATMENT**		
Aspirin	0.912 (0.420, 1.977)	0.815
P2Y12 antagonist	0.976 (0.581, 1.640)	0.928
Anticoagulant	2.564 (1.362, 4.827)	0.004
Statin	0.424 (0.212, 0.848)	0.015
Intensive Statin	0.515 (0.328, 0.809)	0.004
Ezetimibe	1.400 (0.611, 3.204)	0.426
Fibrates	1.189 (0.482, 2.931)	0.707
Omega 3		
Insulin	2.888 (1.531, 5.448)	0.001
Oral antidiabetics	1.081 (0.621, 1.881)	0.783
ACEI	0.474 (0.312, 0.719)	<0.001
ARBs	1.958 (1.247, 3.072)	0.003
Aldosterone antagonist	2.652 (1.564, 4.498)	<0.001
Betablocker	0.815 (0.521, 1.275)	0.370
Nitrates	3.477 (2.146, 5.634)	<0.001
Diltiazem	2.718 (1.100, 6.712)	0.030

Dihydropyridines	1.852 (1.135, 3.023)	0.014
Diuretic	1.997 (1.314, 3.036)	0.001
Proton Pump Inhibitors	2.202 (1.196, 4.054)	0.011
Digoxin	3.472 (0.481, 25.05)	0.217
Amiodarone	3.827 (0.935, 15.66)	0.062

Abbreviations as for table S1.
